# Oral Administration of Fucoidan Can Exert Anti-Allergic Activity after Allergen Sensitization by Enhancement of Galectin-9 Secretion in Blood

**DOI:** 10.3390/biom10020258

**Published:** 2020-02-09

**Authors:** Masashi Mizuno, Kana Sakaguchi, Iwao Sakane

**Affiliations:** 1 Department of Agrobioscience, Graduate School of Agricultural Science, Kobe University, Kobe 657-8501, Japan; cn.tt.346@gmail.com; 2 Central Research Institute, ITO EN, Ltd.; 21 Mekami, Makinohara, Shizuoka 421-0516, Japan; i-sakane@itoen.co.jp

**Keywords:** Anaphylaxis, fucoidan, galectin-9, mast cell, type I hypersensitivity

## Abstract

A previous study revealed that fucoidan inhibited mast cell degranulation through the upregulation of galectin-9 in blood. The purpose of this study is to elucidate its mechanism using ovalbumin (OVA) induced anaphylaxis model mice (BALB/c, Female, 5-week-old) and mast cell line (RBL-2H3 cells). Oral administration of fucoidan after sensitization with OVA/Al(OH)_3_ inhibited reduction of rectal temperature induced by activation of mast cells. Fucoidan increased galectin-9 mRNA expression only in colonic epithelial cells. These results suggested that fucoidan could suppress the allergic symptoms in sensitized mice by inducing galectin-9 production from colonic epithelial cells. In addition, to check the influence of galectin 9 on the degranulation of mast cells, RBL-2H3 cell lines were treated directly with recombinant galectin-9. As expected, galectin-9 inhibited degranulation of RBL-2H3 cells pre-bound with IgE. Moreover, the residual amounts of IgE on RBL-2H3 cells were decreased by an addition of galectin-9. It was demonstrated that galectin-9 could remove IgE even if IgE was already bound to mast cells and suppress the mast cells degranulation induced by antigen. This study shows that fucoidan might become an effective therapeutic agent for patients already developed type I allergic diseases.

## 1. Introduction

Allergy is the abnormal immune reaction against specific substances (allergens) contained in foods, pollens, animal dander, latex, house-dust-mite fecal particle and so on [1]. Allergy can be classified into four types from I to IV, according to difference in pathogenic mechanisms [2,3]. Type I allergy, which is characterized with immediate hypersensitivity reactions, is mediated by interaction between immunoglobulin E (IgE) and antigen resulting in release of mediators including histamine. Development of type I allergy is associated with that of some immune cells, especially mast cells. In the beginning stage, antigens are taken up and processed by antigen-presenting cells (APCs) such as dendritic cells (DCs) [4,5]. The degraded antigens are presented by major histocompatibility complex class II (MHC II) to T cell receptors (TCRs) on naïve CD4^+^ T cells [4,6]. Thus CD4^+^ T cells are activated and develop into type 2 T helper (Th2) cells [6]. Th2 cells secrete Th2 cytokines such as interleukin (IL)-4 and IL-13, which are responsible for class switching of immunoglobulin from IgM to IgE in B cells [7]. Antigen-specific IgE produced by the B cells sensitizes the mast cells by its binding to the high-affinity receptor for IgE (FcεRI) expressed at the surface of mast cells. When the antigen is recognized again by antigen-specific IgE on FcεRI, crosslinking of IgE-FcεRI complexes is caused on mast cells. The complex induces activation of mast cells and then a number of inflammatory mediators, including histamine, lipid-derived mediators, cytokines and chemokines are released from mast cells [7]. These mediators induce allergic reaction, such as tissue swelling, erythema and sneezing [1]. Type I allergic diseases, including food allergy, allergic rhinitis and asthma, are major health problems around the world and patients suffering from these diseases are ever increasing, so that effective therapy for type I allergy is required to cure them.

Galectin-9, which is one of the lectins that binds β-galactoside, is produced in various tissues such as intestine, liver, lung and kidney and so on [8]. Some studies demonstrated that galectin-9 exhibits immune modulation effects. For example, galectin-9 prevented autoimmune arthritis by suppressing the generation of Th17 cells and promoting the induction of regulatory T cells [9]. Galctin-9 also enhanced antitumor immunity by increasing T-cell immunoglobulin and mucin domain 3 (Tim-3)^+^ DCs and CD8^+^ T cells via galectin-9-Tim-3 interaction [10]. Furthermore, it was reported that galectin-9 could suppress the allergic inflammation in a mouse model of allergic asthma [11]. Recently, it has been revealed that galectin-9 could bind IgE and suppress the mast cell activation [12].

Fucoidan, a kind of sulfated polysaccharide obtained from some brown algae, is mainly composed of fucose and sulfated fucose and sometimes contains other sugars, such as mannose, glucose, galactose and xylose [13]. It has been shown that fucoidan exhibited various biological activities, such as antitumor, immunomodulatory [14,15,16], anti-thrombotic [17,18,19] and antioxidant activities [20]. Fucoidan, which is contained in *Saccharina japonica*, is exclusively composed of fucose and sulfated fucose. It was reported that fucoidan is hardly absorbed in intestinal tract and excreted in the feces [17]. A previous study reported that oral administration of fucoidan exhibited anti-allergic effects, whereas its intraperitoneal administration showed no effect [21]. Moreover, it was demonstrated that this anti-allergic activity was due to secrete galectin-9 in blood through intestinal epithelial cells. It was also shown that the intravenous administration of anti-galectin-9 antibody canceled the suppressive effect of fucoidan in the passive cutaneous anaphylaxis (PCA) model. These results suggest that fucoidan might suppress activation of mast cells through promoting production of galectin-9 from intracellular epithelial cells (IECs). Niki et al. reported that recombinant galectin-9 was able to suppress degranulation of a mast cell line, RBL-2H3 cell, not only after but also before IgE stimulation to the cells [12]. Therefore, it was assumed that galectin-9 could bind to IgE on surface of mast cells and suppress the activation of mast cells induced by antigen. 

In this study, the efficacy of fucoidan as therapeutic agent antagonizing type I allergy was examined in ovalbumin (OVA) sensitized mice and investigated in its suppressive mechanism by focusing on the degranulation of mast cells.

## 2. Materials and Methods

### 2.1. Reagents

Eagle’s Minimum Essential Medium (MEM) was purchased from Nissui Pharmaceutical (Tokyo, Japan). Blocking One, Chemi-Lumi One super and Sepasol RNA I super G were purchased from Nakarai Tesque (Kyoto, Japan). Anti-dinitrophenyl (DNP) IgE, *p*-nitrophenyl *N*-acetyl-β-d-glucosaminide, DNP-albumin and Dulbecco’s Modified Eagle’s Medium Nutrient Mixture F-12 Ham (DMEM/F12) were purchased from Sigma-Aldrich (St Louis, MO, USA). Fetal bovine serum (FBS) was purchased from HyClone Laboratories, Inc. (Logan, UT, USA). RPMI 1640 medium was purchased from Life Technologies (Carlsbad, CA, USA). Recombinant mouse galectin-9 was purchased R&D Systems (Minneapolis, MN, USA). Anti-mouse galectin-9 antibody was purchased from Santa Cruz Biotechnology (Dallas, TX, USA). Anti-mouse IgE and anti-goat IgG-HRP antibodies were purchased from Bethyl Laboratories (Montgomery, AL, USA). Other ordinary chemicals and reagents were commercially available with guaranteed quality.

### 2.2. Mice

Female 5-week-old BALB/c mice were purchased from Japan SLC (Shizuoka, Japan). The mice were fed in an air-conditioned animal room at 25 ± 1 °C and acclimated for 7 days before experiments, maintained in filter-top cages in specific pathogen-free conditions in Kobe University Life-Science Laboratory with free access to laboratory chow and water *ad libitum*. All animal experiments were approved and carried out in accordance with the Animal Experiment Ethnics Committee of Kobe University (registration number: 28-11-01).

### 2.3. Cell Culture

Rat basophilic leukemia cell line, RBL-2H3, were cultured in dishes in Eagle’s MEM supplemented with 10% (*v*/*v*) heat-inactivated FBS (57 °C, 30 min), 100 μg/mL streptomycin, 100 U/mL penicillin and 2 mM l-glutamine. Cell cultures were incubated at 37 °C in a 5% CO_2_ incubator and used for the experiments with in passage numbers 17–32. When cells reached 80% confluence, they were recovered from the culture dish or flask by trypsin digestion after washing with PBS. After centrifuging, the cells were suspended in cell media and cultured in a new dish or flask.

### 2.4. Ovalbumin-Induced Allergy Model

Mice were sensitized by 4 times intraperitoneal injection of 300 μL PBS containing 10 μg OVA mixed with 1 mg Al(OH)_3_ adjuvant once in 5 days. Mice were challenged by intravenous injection of OVA (5 μg/mouse) at 7 days after the 4th sensitization and their rectal temperature was measured as the indicator of allergy symptoms using a rectal thermometer for mice (AD-1687; A&D Tokyo, Japan). It has been reported that mast cell released chemical mediator such as histamine by activation and the mediator induced vessel permeability and vasodilatation [22]. Vasodilation has been known to induce hypotension, followed by decrease of body temperature. Thus, decrease of rectal temperature has been commonly used as a marker of systemic allergic symptom [23]. The rectal temperature was measured in 10 min intervals for 90 min.

### 2.5. Oral Administration of Fucoidan to Mice

Fucoidan was extracted from *S. japonica* according to the methods reported by Tanino et al. [21,24]. Fucoidan was dissolved in sterile water and administered to mice intragastrically using an iron probe. Oral administration of fucoidan (60 μg/mouse) was started after the 2nd and 4th sensitizations and continued by the last day of the experiment.

### 2.6. Measurement of Total IgE, OVA-Specific IgE and OVA Specific IgG1

At one day before the injection of OVA/Al(OH)_3_, blood samples were collected from the tail vein. On the final day, whole blood was collected by cardiac puncture. The blood samples were incubated at room temperature for 1 h at 4 °C overnight and were centrifuged at 10,000 rpm for 10 min to obtain the serum. The amount of total IgE, OVA-specific IgE and IgG1 was measured in serum by using BD OptEIA Mouse IgE ELISA Set (BD Bioscience, San Jose, CA, USA), DS Mouse IgE ELISA (OVA) (DS Pharma Biomedical, Osaka, Japan), Anti-Ovalbumin IgG1 (mouse) ELISA kit (Cayman Chemical, Ann Arbor, MI, USA), respectively.

### 2.7. Measurement of Galectin-9

Galectin-9 contents in serum were measured by ELISA assay. Recombinant mouse galectin-9 (10–500 ng/mL, 100 μL/well) or 2-fold diluted samples (100 μL/well) were plated on a 96 well ELISA plate (greiner bio-one, Solingen, Germany) and placed at 4 °C overnight. The wells were washed with PBS containing 0.05% Tween 20 (PBST) and blocked with 1% BSA in PBS for 90 min at room temperature. The primary antibody for galectin-9 (1:100 in PBST) was added after washing with PBST and incubated for 90 min at room temperature. The plate was washed with PBST and secondary anti-goat IgG antibody (1:1000 in PBST) was added and incubated more for 1 h at room temperature. The plate was washed again with PBST and the solution was completely removed. Phosphoric and citric buffer (24.3 mM citric acid, 51.4 mM Na_2_HPO_4_, pH 5.0) containing *o*-phenylenediamine (0.5 mg/mL) and 0.015% H_2_O_2_ (100 μL/well) was added to the plate and subsequently 4N H_2_SO_4_ (50 μL/well) was added to stop the reaction. The absorbance at 492 nm was measured using a microplate reader.

### 2.8. RNA Isolation and Real-Time PCR

Harvested ilium and colon were washed in ice-cold PBS to remove feces and then cut longitudinally. Their IECs were isolated by scraping using a glass slide. Other tissues were lavaged by PBS and frozen using liquid nitrogen. The tissues were cut into small pieces by scissors. Total RNA was extracted from IECs and tissues using Sepasol RNA I super G. The reverse transcription (RT) was conducted using High-Capacity cDNA Reverse Transcription kit (Life Technologies, Carlsbad, CA, USA). The RT reaction was performed in a thermal cycler (Gene Amp^®^ PCR System 9700, Applied Biosystems, Foster City, CA, USA) at 25 °C for 10 min, 37 °C for 120 min and 85 °C for 5 sec. The real-time PCR was performed using a 7500 Fast Real Time polymerase chain reaction (PCR) system (Life Technologies, Carlsbad, CA, USA) using FastStart Universal Probe Master (Rox) (Roche Diagnostics, Basel, Switzerland), in accordance with the manufacturer’s standard protocol. Taq Man probes (Life Technologies) were used in real time PCR and the product numbers were given as follows; mouse Galectin-9 Assay ID: Mm00495295_m1, human Galectin-9 Assay ID: Hs00371321_m1. Mouse β-actin Assay ID: Mm00607939_s1 or human GAPDH Assay ID: Hs99999905_m1 were used as endogenous control. For the relative comparison of mRNA expression levels, the data from real-time PCR were analyzed with a ΔΔCT quantification method and normalized to the amount of β-actin or GAPDH cDNA as an endogenous control.

### 2.9. β-Hexosaminidase Assay

To evaluate the influence of galectin-9 on anti-allergic activity, RBL-2H3 cells were studied in accordance with a previous study [25]. The cells were preincubated with recombinant galectin-9 solutions (5 μg/mL in SB; 119 mM NaCl, 5 mM KCl, 0.4 mM MgCl_2_, 1 mM CaCl_2_, 40 mM NaOH, 25 mM PIPES, 5.6 mM glucose, 0.1% BSA, pH 7.2) for 2 h at 37 °C before sensitization with 1 mg/mL anti-DNP IgE overnight. The cells were challenged with 10 ng/mL DNP-albumin for 10 min at 37 °C. The supernatant (50 μL) was incubated with an equal volume of substrate solution (5 mM *p*-nitrophenyl-*N*-acetyl-β-d-glucosaminide in 0.2 M citrate buffer at pH 4.5) for 1 h at 37 °C. After adding 100 μL/well of stop buffer (0.2 M Tris, pH 8.0), the absorbance at 405 nm was measured using a microplate reader. The percentage of β-hexosaminidase released into the supernatants was calculated as a percentage of the degranulation group (IgE/Antigen group).

### 2.10. Western Blot of IgE Binding Mast Cells

The cells which were sensitized with anti-DNP IgE and then challenged DNP-albumin for β-hexosaminidase assay were applied western blot to measure IgE contents binding mast cells. The cells were washed twice with PBS and lysed in RIPA buffer, containing 150 mM NaCl, 0.5% sodium deoxycholate, 0.1% SDS, 50 mM Tris–HCl (pH 7.4), 50 mM glycerophosphate, 20 mM NaF, 1 mM DTT, 5 mg/mL leupeptin, aprotinin and 100 µM PMSF. Proteins were extracted with 50 μL RIPA buffer and quantified by the Lowry assay. Total proteins (30 μg) were separated by 10 % SDS-PAGE and transferred onto polyvinylidene fluoride membranes (General Electric, Fairfield, CT, USA). Membranes were blocked with Blocking One at room temperature for 1 h and incubated with anti-mouse IgE or anti-β-actin antibodies as the primary antibody at 4 °C overnight, followed by anti-goat or anti-mouse IgG-HRP antibodies as the secondary antibody at 4 °C for 1 h. All signals were detected by enhanced chemiluminescence using Chemi-Lumi One super and the intensity of band was quantified using the Image J program.

### 2.11. Statistical Analysis

Each of the value was expressed as mean ± standard error. Statistical significance between any two groups was analyzed using Student’s *t*-test. Statistical significance between more than two groups was analyzed by one-way ANOVA and Tukey test. Statistical significance was defined as *p* < 0.05 and 0.01.

## 3. Results

### 3.1. Anti-Allergic Effect of Fucoidan in OVA-Induced Allergic Mice

**Figure 1:**
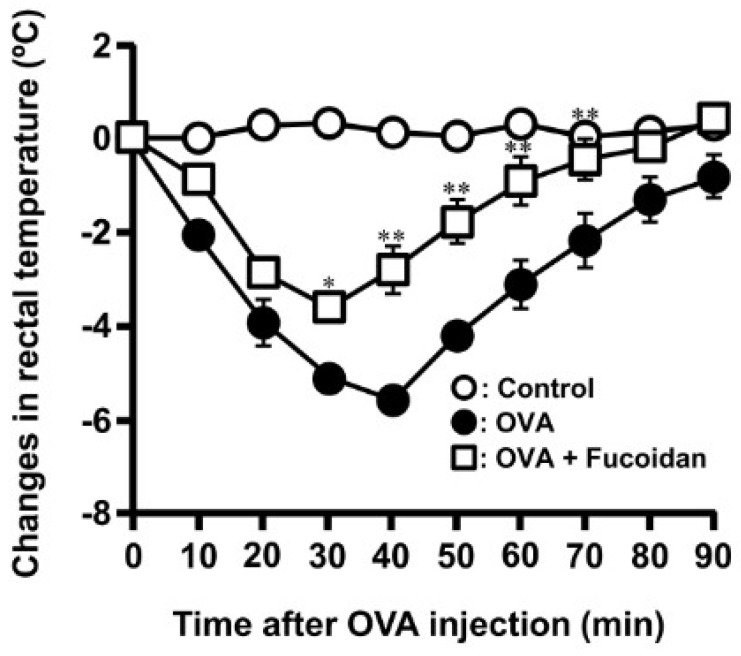
Oral administration of F-fucoidan suppresses the decrease of rectal temperature induced by ovalbumin (OVA) injection. Mice were orally administered F-fucoidan (60 μg/mouse/day) and immunized by injection of OVA and administrated as described in the Materials and Methods. Rectal temperature was measured every 10 min for 90 min immediately following intravenous injection of OVA (5 μg/mouse). * *p* < 0.05, ** *p* < 0.01 significantly different from the values of the group that was not administrated F-fucoidan and injected OVA (OVA control). Values represent means ± SE of 4–5 mice in each group.

It was reported that the oral administration of fucoidan suppressed PCA reaction [21]. To confirm whether fucoidan affected the Th1/Th2 balance, OVA-induced allergic diseases was orally administered with fucoidan from *S. japonica*. Oral administration of fucoidan shows no changes in serum total IgE, OVA-specific IgE and IgG1 to be increased by OVA injection at Day 17 (Table 1). These data suggested that fucoidan could not modulate immune response, particularly Th2 dominant. As it has reported that fucoidan stimulates to secrete galectin-9 which has a high affinity to IgE [12], it was assumed that fucoidan possessed anti-allergic property even if IgE contents did not change. During allergic reactions, mast cells usually undergo degranulation with the release of allergic mediators such as histamine and some proteases. Histamine is carried in the blood to the brain where it stimulates thermoregulatory receptors in the hypothalamus which results in the decrease of body temperature observed during allergic reactions. Measurement of rectal temperature is an easy way of monitoring mice body temperature during allergic reactions. Therefore, the rectal temperature was measured as the other anti-allergic index. As show in Figure 1, the rectal temperature in the sensitization group decreased by −5.6 ± 0.2 °C at 40 min after challenge of OVA compared to control group, whereas in the 200 μg/day fucoidan-treated group, the rectal temperature was only reduced by −3.8 ± 0.3 °C at 30 min and gradually recovered to almost the same level as those of control group. 

### 3.2. Anti-Allergic Property of Fucoidan after Sensitization of OVA

**Figure 2:**
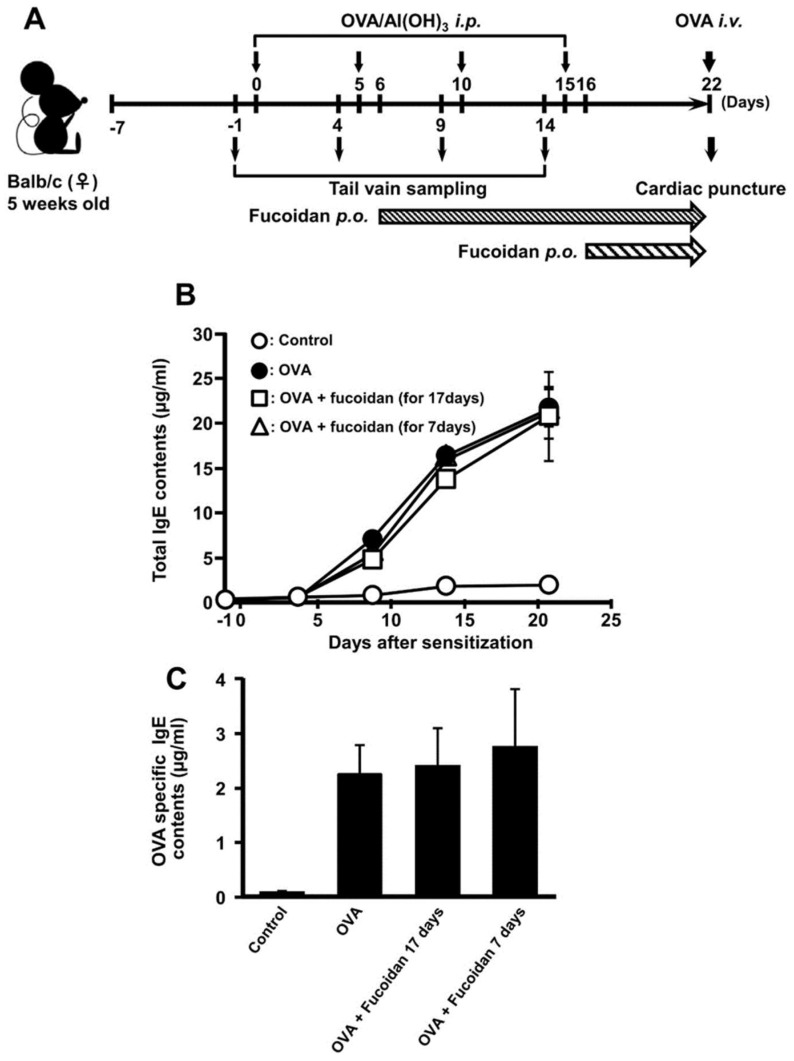
The effect of F-fucoidan on the increase in IgE induced by OVA injection. Mice were immunized by injection of OVA and administrated F-fucoidan orally (60 μg/mouse/day) as described in the Materials and Methods. (**A**) Schedule of OVA-induced allergy experiment. (**B**) Total IgE levels in blood collected from the vain 1 day before OVA injection were measured by ELISA. (**C**) Whole blood was collected by cardiopuncture 1 day after OVA challenge and plasma was obtained. OVA-specific IgE levels in blood plasma were measured by ELISA. Values represent means ± SEM of 4–5 mice in each group.

**Figure 3:**
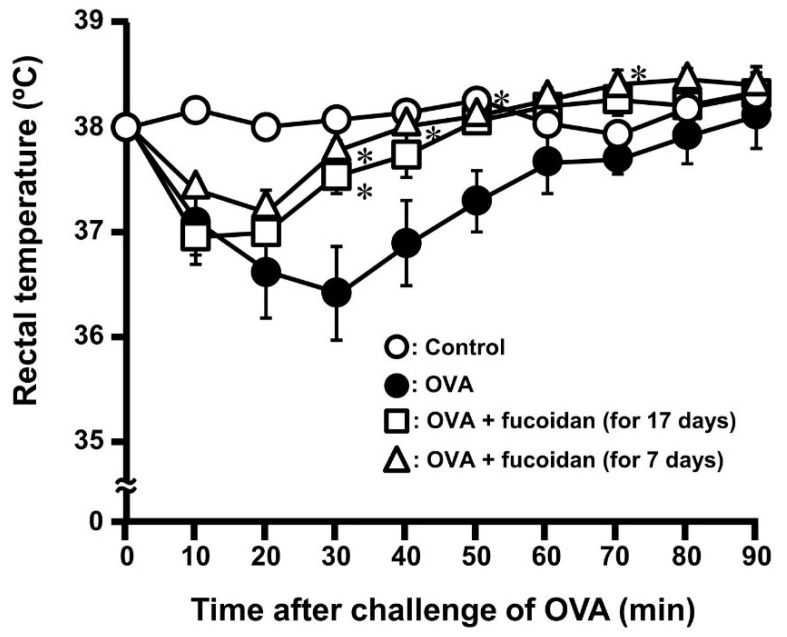
Suppressive effect of F-fucoidan on rectal temperature induced by OVA injection. Mice were immunized by injection of OVA and orally administrated F-fucoidan (60 μg/mouse/day) as described in the Materials and Methods. Rectal temperature was measured as described in Figure. 1. * *p* < 0.05 significantly different from the values of the group that was not administrated F-fucoidan and injected OVA (OVA control). Values represent means ± SEM of 4-5 mice in each group.

It was ascertained that oral administration of fucoidan possesses anti-allergic effect in OVA-induced allergic mice without influence of IgE contents (Figure 1). Moreover, it was demonstrated that galectin-9 secretion in blood plasma was contributed to exert anti-allergy activity in PCA reaction [21]. It was revealed that galectin-9 could have a high-affinity with IgE and suppress the mast cell activation [12]. Therefore, we hypothesized that fucoidan can fulfil anti-allergic activity after the sensitization of mast cells. As shown in Figure 2A, oral administration of fucoidan was carried out a day after 2nd and 4th sensitization which were corresponding to the period of fucoidan administration for 17 days and 7 days, respectively. Although total IgE and OVA-specific IgE contents increased with the frequency of sensitization, fucoidan administration did not affect predictably their contents (Figure 2B,C). However, a rectal temperature shows the drastic inhibition of decrease by oral administration of fucoidan after OVA sensitization (Figure 3). The rectal temperature at 30 min after challenge of OVA in the sensitization group decreased by −1.54 ± 0.40 °C, whereas in fucoidan-treated group after 17 and 7 days OVA sensitization, the rectal temperature was only reduced by −0.48 ± 0.12 °C and −0.25 ± 0.08 °C, respectively. Thus, it was ascertained that oral administration of fucoidan possessed anti-allergic property after sensitization.

### 3.3. Glectin-9 Contents in Blood and Lgals9 mRNA Expression in Tissues by Administration of Fucoidan after OVA-Sensitization

**Figure 4:**
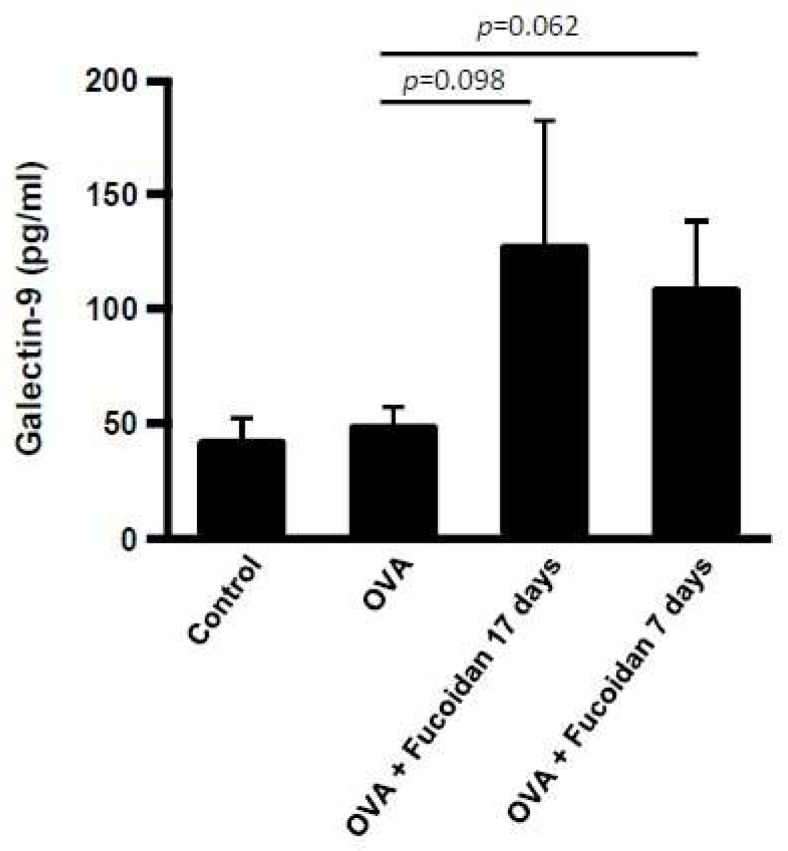
Galectin-9 level in blood plasma of mice administered F-fucoidan. Mice were immunized by injection of OVA and administrated F-fucoidan orally (60 μg/mouse/day) as described in the Materials and Methods. Whole blood was collected by cardiopuncture 1 day after OVA challenge and plasma was obtained. The concentration of galectin-9 in blood plasma was measured by ELISA. Values represent means ± SEM of 4−5 mice in each group.

**Figure 5:**
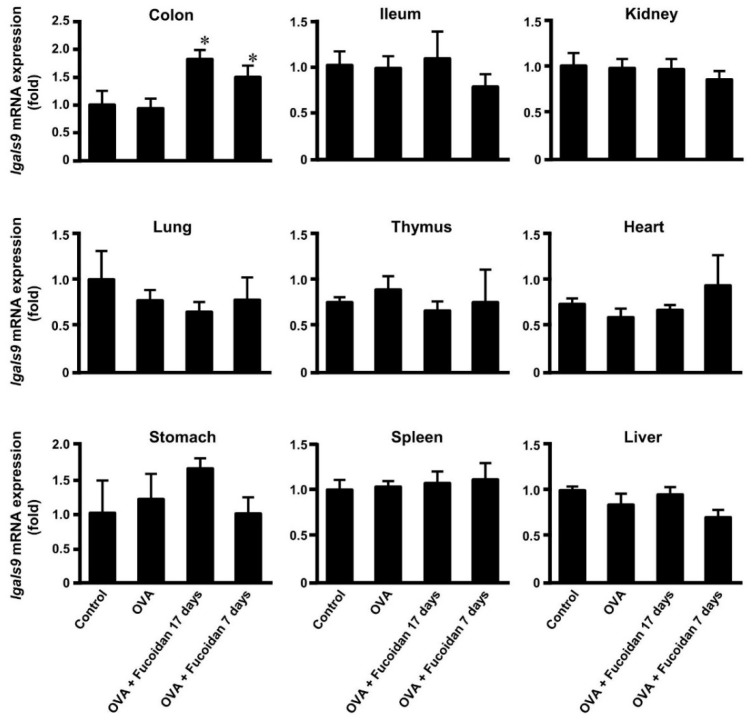
*lgals9* mRNA expression in some tissues.Galectin-9 mRNA expression levels in colon, ileum, kidney, lung, thymus, heart, stomach, spleen and liver of mice after measurement of rectal temperature were measured. * *p* < 0.05 significantly different from the values of group that was injected OVA and not administrated F-fucoidan (OVA control). Values represent the means ± SEM of 4−5 mice in each group.

As shown in Figure 3, it was clear the administration of fucoidan after sensitization of OVA shows anti-allergic activity. Previous studies showed that galectin-9 level in blood plasma increased by administration of fucoidan [21]. To examine the relation between anti-allergic effect of fucoidan and galectin-9 secretion, galectin-9 level in blood plasma was measured. As shown in Figure 4, the galectin-9 level was not affected by OVA injection. On the other hand, the tendency to increase galectin-9 level was observed when fucoidan was administered for 17 days and 7 days after OVA-sensitization. Galectin-9 is widely distributed in various tissues [26] and a large variety of cells including epithelial cells, endothelial cells and immune cells produce galectin-9 [27,28,29]. As the administration of fucoidan secreted galectin-9 in blood, the galectin-9 mRNA (*lgals9*) levels of tissues indicated in Figure 5 were measured. Though *Lgals9* levels were increased only in colon by oral administration of fucoidan after 2nd and 4th sensitization, no drastic differences recognized in the other tissues. These results suggested that IECs, especially colonic epithelial cells might be responsible for production galectin-9 responded to fucoidan.

### 3.4. Effect of Galectin-9 on Degranulation of Mast Cells

**Figure 6:**
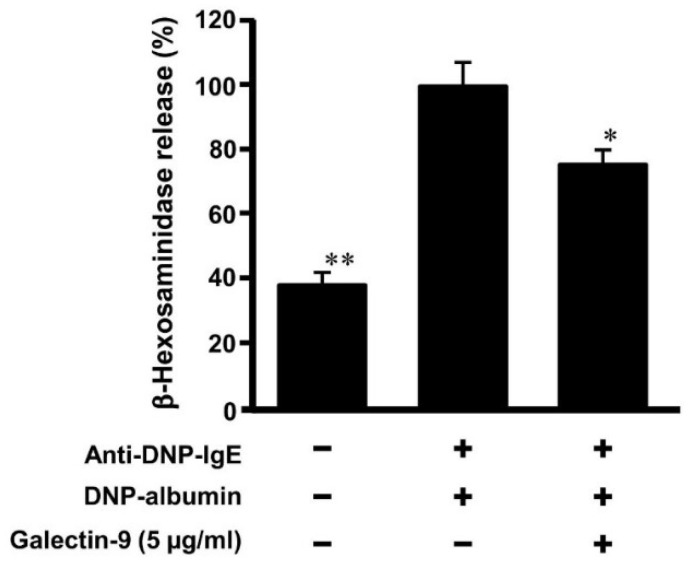
Galectin-9 suppresses the degranulation of mast cells. RBL-2H3 cells were incubated with anti DNP-IgE (1 μg/mL) overnight and then cells were incubated with or without recombinant galectin-9 for 2 h. Degranulation of RBL-2H3 cells was evoked by DNP-albumin as an antigen. The percentage of β-hexosaminidase release was calculated as a percentage of the group added IgE without galectin-9. * *p* < 0.05, ** *p* < 0.01 significantly different from the values of group that was added IgE and incubated without recombinant galectin-9. Values represent the means ± SEM (*n* = 3).

OVA-induced allergic model in Figure 3 and Figure 4 suggested that oral administration of fucoidan might suppress the activation of mast cells by galectin-9 secretion in blood plasma under allergy onset. To ascertain the inhibition of galectin-9 in mast call degranulation by antigen after the sensitization of IgE, RBL-2H3 cells were treated with a recombinant galectin-9 after the sensitization of anti DNP-IgE. The treatment of recombinant galectin-9 (5 μg/mL) suppressed to approximately 75% of β-hexosaminidase release from RBL-2H3 induced by DNP-albumin as the antigen (Figure 6). Although it was reported that galectin-9 promoted the apoptosis in various cells including mast cells [30], MTT assay shows no influence on cell viability at less than 10 μg/mL concentration of galectin-9. These results indicated that the treatment with galectin-9 to the sensitized mast cells inhibited their degranulation by antigen challenge.

### 3.5. Affinity of Galaectin-9 to Mast Cells against IgE

**Figure 7:**
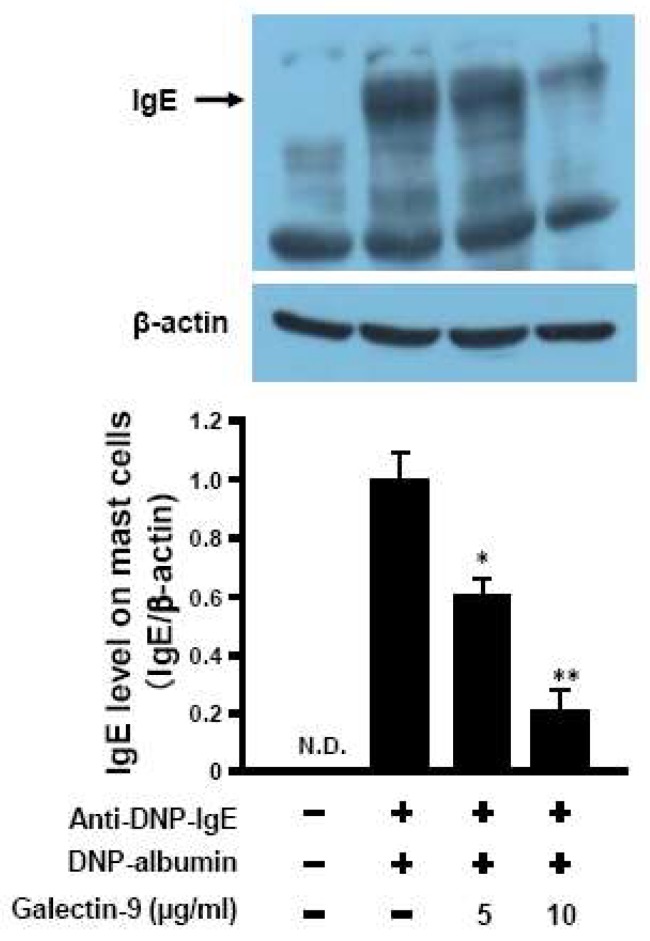
Galectin-9 decreases IgE contents on mast cells. RBL-2H3 cells were incubated with anti DNP-IgE (1 μg/mL) overnight and then incubated with or without recombinant galectin-9 for 2 h. The cells were collected and lysed to obtain the proteins. IgE level in the samples were measured by western blotting analysis. * *p* < 0.05, ** *p* < 0.01 significantly different from the values of group that was added IgE and incubated without recombinant galectin-9. Values represent the means ± SE (*n* = 3).

It has reported that galectin-9 could have a high-affinity with IgE and suppress the mast cell activation [12]. Galectin-9 secreted in blood by F-fucoidan administration might possess the property to replace IgE which was already bound on mast cells. To elucidate this hypothesis, IgE contents bound with RBL-2H3 cells which was pre-incubated with anti DNP-IgE overnight and then incubated with galectin-9 were detected by western blot. As shown in Figure 7, the amount of IgE bound to RBL-2H3 decreased in a dose-dependent manner by the recombinant galectin-9. The IgE bound to the mast cells incubated with galectin-9 at the concentration of 5 and 10 μg/mL decreased to 36.7% and 76.6%, respectively.

## 4. Discussion

The reaction of type I allergy is sorted 2 phase; induction phase and effector phase. In induction phase, antigen presentation induces differentiation of naïve helper T (Th) cells to Th2 cells and Th2 polarization promotes antigen-specific IgE production by B cells. The increased IgE binds FcεRI on mast cells in inflamed site. In effector phase, antigen-sensitized mast cells are activated via cross-linking of IgE-FcεRI by re-invasion of antigen. Therefore, it is predicted to be important for suppression of allergic symptoms that Th2 polarization and IgE generation are inhibited or the release of inflammatory mediators is decreased directly by suppression of mast cells activation.

A number of studies reported that some food factors including flavonoids [31], fatty acids [32] and polysaccharides [33] exhibited anti-allergic effects. Fucoidan, dietary polysaccharide obtained from brown sea algae, had anti-allergic effects by altering Th1/Th2 balance and preventing IgE production from B cells when it was intraperitoneally administered [29,34,35]. However, there are few reports which indicate the effects of oral administration of fucoidan on allergic symptoms.

Previous studies showed that a continuous oral administration of F-fucoidan from *S. japonica* prior to the sensitization to mice moderated allergic symptoms by suppression of mast cells activation, whereas the oral administration of F-fucoidan did not affect IFN-g and IL-4 productions from splenocyte and the levels of total IgE and OVA-specific IgE in blood [21]. As shown in Figure 2 and Figure 3, the oral administration of F-fucoidan to mice already sensitized with OVA could suppress rectal temperature with no influence in the total IgE and OVA-specific IgE levels in blood plasma as well as the previous report. Moreover, it was revealed that the anti-allergic effect of F-fucoidan was not observed when mice were intraperitoneally administrated F-fucoidan. Accordingly, F-fucoidan was interacted with the intestinal tract to exert its anti-allergic effect. As shown in Figure 5, galectin-9 mRNA increased only on colonic epithelial cells by F-fucoidan administration, suggesting that colonic epithelial cells might possibly influence production of galectin-9 in response to F-fucoidan.

It was reported that galectin-9 bound to IgE with high affinity and suppressed the degranulation of mast cells [12]. This suppression in mast cells degranulation was caused when the cells were incubated with galectin-9 not only before but also after IgE addition. In this study, mast cells were once sensitized with IgE, washed to remove free IgE and then incubated with galectin-9 to test if IgE once attached on mast cells was antagonized by galecton-9 to reduce the degranulation. As the results shown in Figure 6, galectin-9 suppressed degranulation of sensitized mast cells. Furthermore, the level of IgE attached on mast cells was decreased when sensitized mast cells were incubated with galectin-9 (Figure 7). The results suggested that galectin-9 could remove IgE from the mast cells and hence the mast cells activation was suppressed.

Galectin-9 mRNA in IECs and serum galectin-9 levels increased by dietary supplementation with galacto- and fructo-oligosaccharides and *Bifidobacterium breve* M-16V and the increase was correlated with the prevention of allergic symptoms in mice [36]. In this study, galectin-9 mRNA in colonic epithelial cells of OVA-sensitized mice only increased after the treatment with F-fucoidan, although OVA administration did not influence the galectin-9 mRNA (Figure 5). Similar trend was observed in galectin-9 level in blood plasma (Figure 4). The present study demonstrated that F-fucoidan enhanced the production of galectin-9 in colonic epithelial cells, which suppressed the activation of the mast cells through decrease in IgE attached on mast cells. These findings suggest that galectin-9 induced by F-fucoidan could restore the mast cells sensitized by allergens and that F-fucoidan could have a promising potential as a therapeutic agent for patients with allergic diseases.

## Figures and Tables

**Table 1:** Effect of F-fucoidan on total IgE, OVA-specific IgE and OVA-specific IgG_1_ contents in OVA sensitized mice.

	Control	OVA	OVA + Fucoidan
Total IgE (μg/mL)	0.80 ± 0.16	10.64 ± 1.28	11.52 ± 1.51
OVA specific IgE (ng/mL)	N.D.	661.26 ± 140.81	550.73 ± 70.04
OVA specific IgG_1_ (μg/mL)	N.D.	882.45 ± 245.75	1203.28 ± 162.13

## Abbreviations

APCs, Antigen-presenting cells; DCs, dendritic cells; DNP, dinitrophenyl; ELISA, Enzymed-linked immunocorbent assay; FcεRI, High-affinity IgE receptor; IECs, intracellular epithelial cells; IgE, Immunoglobulin E; MEM, Eagle’s minimum essential medium; OVA, Ovalbumin; PBS, Phosphate buffered saline; PBST, Phosphate buffered saline with Tween 20; PCA, passive cutaneous anaphylaxis; TCRs, T cell receptors; Th2, Type 2 T helper; Tim-3, T-cell immunoglobulin and mucin domain 3.
